# Effects of Earthworms and Agricultural Plant Species on the Soil Nematode Community in a Microcosm Experiment

**DOI:** 10.1038/s41598-019-48230-0

**Published:** 2019-08-12

**Authors:** Xinli Niu, Peipei Zhai, Weixin Zhang, Yanfang Gu

**Affiliations:** 1 School of Life Sciences, Henan University, Kaifeng, 475004 China; 2 College of Environment and Planning, Henan University, Kaifeng, 475004 China

**Keywords:** Agroecology, Agroecology

## Abstract

Both earthworms and plants may affect the soil nematode community. However, the effects of earthworms and plant species interactions on soil nematode community are poorly understood. We explored how an epigeic earthworm *Eisenia fetida* affects the soil nematode community in systems with three representative plants (wheat, cotton and cabbage) which were grown in pots with or without added earthworms under greenhouse conditions. Earthworm presence decreased the abundance of total nematode and all four nematode trophic groups, except for the fungivore and predator/omnivore nematodes in wheat systems, but increased the genus richness of nematode in all treatments. Due to plant identity and different root exudates, plants had significant effects on soil nematode abundance. Compared with the no plant and without earthworm treatment, wheat and cabbage had the higher stimulation of the abundance of total nematode, bacterivores and fungivores, and cotton had the higher stimulation of the abundance of fungivores and predators-omnivores; whereas earthworm presence mostly weakened the stimulation effects of plant species on soil nematode abundance which indicated earthworms had the enhanced effects in the presence of plants. The interaction affected soil nematode abundance (total nematodes, bacterivore, fungivore and omnivore-predators) and community diversity indices (diversity index *H*′, evenness index *J*′, community maturity index ∑MI, Simpson dominance index λ and nematode channel ratio NCR). Principal component analysis showed that plant species affected soil nematode community composition. Redundancy analysis indicated plant species and biomass accounted for 41.60% and 34.13% of the variation in soil nematode community structure, respectively; while earthworms explained only 6.13%. Overall, current study suggest that earthworm could inhibit nematode abundance; whereas, plants have exerted greater influences on nematode community structure than earthworm presence due to their species-specific effects on different trophic groups of nematodes.

## Introduction

Soil nematodes are an important part of soil biota and occupy positions at the primary, secondary and tertiary consumer levels in soil food webs^1–3^. As the most abundant and diverse type of soil invertebrate comprising all major trophic groups, soil nematodes may represent the complexity of soil food webs^4^. Nematodes can serve as model soil invertebrates that provide a holistic measure of the biotic and functional statuses of soils^5^. Living in soil microecosystems, nematode communities are sensitive to environmental changes and are therefore considered useful bioindicators for soil health assessments^6,7^.

Earthworms represent the largest component of the soil animal biomass and are commonly known as ‘ecosystem engineers’^8^. Previous studies have shown that endogeic and anecic earthworms have negative, positive or neutral effects on soil nematodes in different ecosystems^9–13^. In addition, reports have indicated that the epigeic earthworms have effects on soil nematode populations. Studies have found that epigeic earthworm suppressed bacterivorous nematode numbers (more than 50%) in fresh manures and sludge wastes^14^ and 30% in pig manure^15^. However, the epigeic earthworms could also increase the number of plant-parasitic nematodes in maize with earthworms by 27% compared with maize without earthworms^16^. In addition, in peat meadow soil, compared to the control (without earthworms), the introduction of epigeic earthworms (*Lumbricus rubellus*) decreased the total number of nematodes after 30 days, while 120 days after earthworm addition, the total nematode numbers showed no significant difference^17^. These results indicated that the effects of epigeic earthworms depended on the soil or substrate systems, nematode trophic groups or even the duration of earthworm presence. Most studies have focused on substrate conditions such as those in manure, sludge or peat meadow soil; whereas, few studies^18–20^ have focused on the effects of earthworms on soil nematode community under planting crops.

In addition, there have been considerable studies in how plants affect the abundance and diversity of soil nematodes^21–23^. Studies have demonstrated that different plant species could affect the numbers of plant-feeding nematodes, the nematode taxonomic diversity and the ratio of fungal to bacterial plus fungal feeders^24^ although they did not affect the omnivore-predators feeding groups^25–27^. Reasons may be due to the quantity and quality of organic matter produced by different plant species^28^, as well as the different harmful substances exuded by certain plant species for plant-feeding nematodes^29^ and the different amounts of bacteria populations or plant species modify nematode community in bacterivorous nematodes^30^. Wheat, cotton and cabbage are the main agricultural crops in northern China. Different plant species may produce specific root exudates which may result in a stimulation or inhibition on specific trophic group of nematodes.

Although the effect of earthworms on plant growth is well recognized, the combined effects of plants and earthworms on soil nematodes have not been well investigated. Study indicated plants could modify the effects of earthworm on the soil microbial community and its activity^31^. Whether the effect of earthworms on soil nematode community are more significant than that of plant species has not been clarified. The effects of earthworms on soil nematodes may be reduced or enhanced by plants. The combined effects of earthworms and plant species can be expected to affect soil nematode communities in different ways. A laboratory research can be an effective way to research these effects and elucidate novel aspects of interactions among plants, earthworms and nematodes. In the current study, we hypothesized: (1) that earthworm presence may decrease nematode abundance under planting crops, and such effect may be enhanced in a system where earthworms stimulated plant growth; (2) that either plant species or earthworms may have a more significant effect on the soil nematode community composition; (3) that the interactive effects of earthworms and plant species would influence soil nematode abundance and community structure.

## Materials and Methods

### Microcosms

A sandy mineral soil (5–20 cm below the surface) from ordinary farmland (continuous cropping winter wheat in October, every year) collected at Henan University was used in the experiment. The soil was sieved (2 mm mesh). Maize straw (C/N = 52) was sundried and milled to pass through a 2 mm diameter mesh before use. 40 kg soil (dry weight) and 2400 g maize powder (earthworm food) were mixed thoroughly, and divided into 80 pots (diameter 12 cm and height 15 cm) (500 g soil + 30 g maize powder/pot). Other soil fauna, including macrofauna was manually removed. The starting soil contained approximately 0.08 g/kg total nitrogen, 0.38 g/kg organic carbon, and 1.58 g/kg total carbon (C/N ratio 21.07) and had a pH of 8.08.

*Eisenia fetida* is an easily cultured, and has a broad distribution in China. The gut contents of 80 earthworms were emptied on moistened tissue paper for 24 h at room temperature before the experiment, and these earthworms (fresh weight 0.32 ± 0.02 g/earthworm) were added into 40 pots (2 earthworms/pot). Another 40 pots, with 10 no plant pots and 30 pots with plants, were earthworm free. The earthworm numbers corresponded to a density of 216 individuals/m^2^, which was observed in winter wheat fields^32^. To prevent the earthworms from escaping, 80 gauzes (height 50 cm, diameter 15 cm) which would affect the growth of plants were wrapped around the pots.

Seeds of winter wheat cultivar Zhoumai 18 (Plant Genetic Resources and Genetic Engineering Laboratory, Henan University), cotton cultivar Zhongmian 79 (State Key Laboratory of Cotton Biology, Henan University), and cabbage cultivar Zhonggan 11 (Vegetable and Flower Research Institute, Chinese Academy of Agricultural Sciences) were surface sterilized with sodium hypochlorite (1%), sown on wet paper in Petri dishes and placed in a climate chamber (14 h/10 h, light/dark, 25 °C). Germinated plant seeds (one cotyledon in wheat; two cotyledons in cotton and cabbage) were transplanted into every pot. Earthworms were added after the plants had grown for one week. All 80 pots were placed in five blocks on greenhouse benches, and each block contained 2 replicates (pots) of the 8 treatments. The blocks were redistributed randomly in the greenhouse every week. The experiment began on December 3^rd^ in 2015. No fertilizers were applied during the experiment, and other weed seedlings germinating from seeds in the soil were removed. The soil was maintained at 70% soil field capacity (checked via regular weighing of the pots).

### Experimental design

The experiment was conducted in a greenhouse in Henan University, China, and included two factors: plant species and earthworm treatments. Earthworms were added or not under the no plant treatment and three different agricultural plant species treatments, resulting in 8 treatments. Each treatment was replicated ten times, resulting in 80 microcosms. Control microcosms consisted of no plant and each species of plant without earthworms. No plant, wheat, cotton, and cabbage systems were grown in greenhouses for 15 weeks under the following controlled conditions: relative humidity = 60–85%, temperature = 23–25 °C, light intensity = 600 μmol/m^2^/s and photo period = 14 h.

### Harvesting plants, soil sampling and analysis

After 15 weeks, all plants were cut at ground level and separated into two parts: shoot biomass and root biomass. The shoot dry weight was determined after drying for 72 h at 70 °C. We only analyzed samples in which the number of earthworms found was the same as the number of earthworms introduced. In order to keep the same effective replicates in all the treatments, 3 replicates were eliminated for the no plant, wheat, cotton, and cabbage treatments. After cleaning, the roots were dried at 60 °C for 72 h and weighed. The fresh soil samples were stored in a refrigerator at 4 °C for subsequent soil analysis and nematode extraction.

The analyzed soil properties included the soil pH (H_2_O), total carbon (TC), total nitrogen (TN), organic carbon (SOC), and the ratio of TC to TN (C/N). Soil pH was measured in a 1:2.5 soil-distilled H_2_O suspension using a glass electrode (Sartorius PB-10). TC, TN and SOC were analyzed using an element analyzer (Vario MACRO cube, Elementar Inc., Germany).

### Soil nematode extraction and identification

Nematodes were extracted from 50 g fresh soil from each pot by using the modified Baermann method^33^. Another 50 g fresh soil was used to determine the soil water content. Soil water content was measured gravimetrically using soil sample from each pot dried at 105 °C for 24 h. The extracted nematodes were preserved in TAF fixation (40% formaldehyde 7 ml, triethanolamine 2 ml, and distilled water 91 ml)^34^. Nematode abundance was measured as individuals per 100 g dry soil, and after counting the total number of nematodes, 100 nematode individuals from each sample were identified to the genus level according to Bongers^35^ using an optical microscope (Motic, BA210, Motic Corporation). For samples in which there were fewer than 100 nematodes, all specimens were identified. The soil nematodes were assigned to four trophic groups: bacterivores (Ba), fungivores (Fu), plant parasites (Pp), and omnivores-predators (Om) with their corresponding colonizer-persister (cp) groups^36,37^.

### Soil nematode community analysis

The following nematode community indices were calculated: (1) dominant genera (Relative abundance > 10%, dominant genera; 1% < relative abundance < 10%, common genera; relative abundance < 1%, rare genera); (2) species richness (SR); (3) the Shannon-Wiener diversity index (*H*′); (4) the modified maturity index (ΣMI); (5) evenness (*J*′); (6) Simpson’s index for dominance (λ); and (7) the nematode channel ratio (NCR)^7,38^.

Margalef richness index, ${\rm{SR}}=\frac{S-1}{lnN}$

Shannon-Wiener diversity index, $H^{\prime} =-\mathop{\sum }\limits_{i=1}^{S}\,Pi\times lnPi$

The modified maturity index, $\sum {\rm{MI}}={\sum v(i)}^{cp1-5}\times f{(i)}^{cp1-5}$

Evenness index, $$\,J^{\prime} =\frac{H^{\prime} }{lnS}$$

Simpson dominance index, $\,{\rm{\lambda }}=\sum P{i}^{2}$

Nematode channel ratio, ${\rm{NCR}}=\frac{{\rm{B}}}{{\rm{B}}+{\rm{F}}}.$

In the above equations, ‘*S*’ is the total number of nematode genera in the community, ‘*N*’ is the total number of nematodes in the community, ‘*Pi*’ is the proportion of the individuals of “*i*th” group in the community, ‘$v(i)$’ is the c-p value of “*i*th” taxon, ‘$f(i)$’ is the frequency of *“i*th” taxon, and ‘B’ and ‘F’ are the numbers of bacterivores and fungivores in the total soil nematode population. SR, *H*′ and *J*′ indices are calculated and used as an indication of the diversity of the soil nematodes. Lower and higher values of $\sum {\rm{MI}}$ indicate disturbed and stable nematode communities, respectively. The higher λ reflects the more uneven distributions of different genera in soil nematode community and the lower diversity of the soil nematodes. The NCR is a powerful index to assess the decomposition pathway of soil matter and might indicate the contribution of bacteria and fungi to the rate of mineralisation.

### Statistical analyses

Results are shown as the means ± SE. Data normality was checked to ensure that the data distribution met the underlying assumptions for further statistical analysis. If necessary, nematode abundance and generic richness were ln(x + 1) transformed. The percentage of trophic groups of soil nematode was arcsine-transformed before two-way analysis. Paired t tests were used to compare the difference (P < 0.05) of plant biomasses between with and without earthworm addition treatments under each plant species. Two-way analysis of variance was performed to test the effects of earthworms and plant species on soil characteristics, nematode total genera richness, nematode abundance, relative nematode abundance and ecological indices. Significant differences in the main effects were further analyzed by paired comparison with the Tukey HSD test. All statistical tests were conducted using the SPSS version 19.0 statistical software (SPSS Inc., Chicago, IL, USA).

Principal component analysis (PCA) in CANOCO version 4.5 was used to measure the soil nematode community composition according to the relative abundances of nematodes^39^ in the absence or presence of earthworms under the no plant treatment and three different plant species treatments. A total of 56 PCAs were analyzed. Each PCA represented a microcosm. Redundancy analysis (RDA) in CANOCO version 4.5^40^ was performed to explore the nematode community composition in relation to environmental factors (plant biomass, plant species, with or without added earthworms, soil properties). Interpretation proportions of environmental factors were calculated by using manual selection referring to a reference^23^. Monte Carlo permutation tests were conducted using 499 random permutations in order to determine the statistical significance of the first and all ordinations axes.

## Results

### Earthworm and crop biomasses

At the end of the experiment, 56 earthworms (0.34 ± 0.01 g/per earthworm) were obtained. Plant biomasses were shown in Table 1. Paired *t* tests indicated earthworm presence significantly increased the shoot biomass of wheat and cotton.

**Table 1:** Biomasses (mean ± SE, n = 7) of 3 different agricultural plant species.

Biomass (dry weight g)	Wheat		Cotton		*Cabbage*	
	−*E. fetida*	+*E. fetida*	−*E. fetida*	+*E. fetida*	−*E. fetida*	+*E. fetida*
Shoot biomass	0.54 ± 0.09	0.98 ± 0.14*	0.76 ± 0.12	1.49 ± 0.12**	2.44 ± 0.22	2.60 ± 0.20
Root biomass	0.22 ± 0.11	0.30 ± 0.03	0.37 ± 0.14	0.44 ± 0.08	0.24 ± 0.04	0.27 ± 0.03

Paired *t* tests, ns, not significant; **Significant at P < 0.01; *Significant at P < 0.05.

### Soil nematode community composition

Forty nematode genera were identified during the study (Table 2). The two most abundant feeding groups were bacterial feeders and plant parasite nematodes. In the absence of earthworms, the dominant genera *Cephalobus* and *Rhabditis* were identified in no plant and wheat; *Cephalobus* and *Tylenchus* which has been classified as “plant associated”^41^ were identified in cotton; and *Cephalobus*, *Acrobeles* and *Rhabditis* were identified in cabbage. However, in the presence of earthworms, the dominant genera *Cephalobus* was found in no plant and cotton; *Aphelenchoides* and *Aphelenchus* were in wheat; and *Cephalobus* and *Tylenchus* were in cabbage (Table 2).

**Table 2:** Proportional contributions (%) of various genera to the nematode assemblages in the absence or presence of  earthworm samples derived from no plant and three different plant species (mean, n = 7).

Genus	c-p	No plant		Wheat		Cotton		Cabbage	
		−*E. fetida*	+*E. fetida*	−*E. fetida*	+*E. fetida*	−*E. fetida*	+*E. fetida*	−*E. fetida*	+*E. fetida*
**Bacterivores**									
*Cephalobus*	2	18.43	12.29	13.00	9.86	13.14	12.14	12.71	14.00
*Eucpehalobus*	2	3.86	3.14	4.57	3.29	3.00	5.14	4.43	6.00
*Acrobeloides*	2	0.43	1.43	1.29	1.29	0	0	2.50	3.86
*Chiloplacus*	2	4.00	5.57	3.29	1.86	0.57	1.86	4.50	4.86
*Cervidellus*	2	1.86	2.57	7.86	3.86	2.00	1.71	2.00	3.43
*Acrobeles*	2	2.14	2.71	5.43	2.00	4.43	3.43	11.86	2.43
*Rhabditophanes*	1	0	0	0	0	0	0	0	1.43
*Caenorhabditis*	1	0	3.71	4.43	1.00	0	0	0	0.29
*Rhabditis*	1	12.14	8.43	11.43	3.71	10.00	8.71	11.43	7.43
*Mesorhabditis*	1	0	0	0	0	0.14	0.57	0	0.57
*Prismatolaimus*	3	1.43	1.71	0	0	0	0	1.00	1.14
*Brevibucca*	2	0.86	0.86	0.29	0.71	0	0	0	0
*Protorhabditis*	1	0	3.86	3.71	1.57	0	0	0	0
*Placodira*	2	0	0	1.29	1.00	0	0	0	0
*Plectus*	2	1.86	2.14	0	0	0	0	0.29	0.86
*Alaimus*	4	2.00	3.00	2.57	0.43	0	0.43	0	0.29
**Fungivores**									
*Aphelenchoides*	2	2.71	3.57	5.14	10.57	9.00	7.00	6.07	3.86
*Tylencholaimus*	4	0.29	1.14	1.57	1.57	2.00	3.57	1.18	0.86
*Aphelenchus*	2	5.00	3.43	5.00	12.00	7.86	7.43	9.86	6.29
**Plant parasites**									
*Cephalenchus*	3	10.86	8.14	3.43	5.57	1.86	3.86	8.00	9.14
*Tylenchus*	2	4.57	4.43	5.57	8.86	11.00	9.00	8.14	10.71
*Psilenchus*	2	0.43	0.43	0	0	4.86	5.43	3.00	3.71
*Pratylenchus*	3	12.14	8.00	2.29	3.57	0	0	0.71	1.14
*Helicotylenchus*	3	2.29	2.57	3.71	5.00	3.14	3.86	2.89	5.14
*Rotylenchus*	3	2.00	2.71	0	0	3.71	3.43	3.64	5.14
*Xiphinema*	5	0.29	1.00	1.43	1.29	1.57	3.29	0.14	0.29
*Paratylenchus*	2	0.14	0.85	1.57	1.00	0	0.57	0	0
*Oxydirus*	5	0	0	0	0	0	0.71	0	0
*Longidorus*	5	1.71	1.43	1.00	1.29	0	1.57	0	0
*Nothotylenchus*	2	0.86	1.86	1.57	4.00	4.29	5.43	1.29	1.86
**Omnivores-predators**									
*Dorylaimus*	4	2.00	1.14	0.86	2.43	3.00	2.29	0.71	0.43
*Eudorylaimus*	4	0.71	0.71	0.57	0.71	5.29	1.00	0.86	0.57
*Enchodelusthorne*	4	0.57	1.00	0.71	1.29	0	0	1.14	1.14
*Mesodorylaimus*	5	0.29	0.57	1.00	1.57	0.43	0.86	0	0
*Mononchus*	4	0	0	0	0	0	0.29	0	0
*Labronema*	5	1.14	1.57	1.29	2.29	3.43	2.43	0.14	0.71
*Discolaimus*	4	0	1.00	1.00	1.71	0	0	0.75	1.14
*Aporcelaimus*	5	1.86	1.86	2.00	4.43	5.00	3.57	0.75	1.29
*Thorneella*	4	1.14	1.14	0.71	0.43	0.29	0.43	0	0
*Doryllium*	4	0	0	0.57	0.57	0	0	0	0

### Nematode abundance and the relative abundance of trophic groups

**Figure 1:**
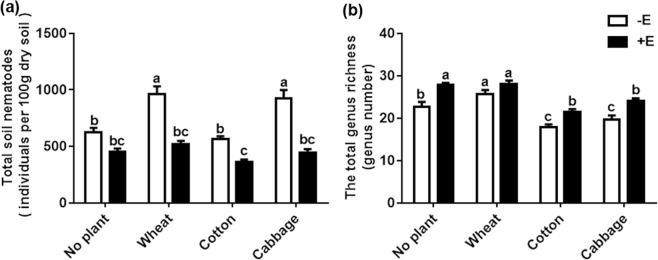
Total abundance of soil nematodes (individuals per 100 g dry soil) (**a**) and total genera richness (**b**) under no plant and three plant species with or without added earthworms. All valued are means + SE, n = 7. Differences letters indicate significant differences between treatments (Tukey HSD test).

Earthworm addition significantly suppressed the abundance of total soil nematodes in three different plant species (Fig. 1a), when compared with no addition of earthworm, which showed the significant interaction between plant species and earthworms (Table 3). Compared to without earthworm treatments, earthworm additions significantly increased the total genus richness in all treatments except for in wheat. However, no significant interaction between earthworms and plant species was observed in total genus richness (Fig. 1b and Table 3).

**Table 3:** Changes in the values of the total genus richness of soil nematode (genus number), and nematode abundance (individuals per 100 g dry soil) under no plant and three plant species with or without added earthworms, and two-way ANOVA for effect of earthworms, three different agricultural plant species and interaction of earthworms × plant species on the total genus richness, and the abundance of soil nematodes.

SN	No plant		Wheat		Cotton		Cabbage		Results of two-way ANOVA					
	−*E. fetida*	+*E. fetida*	−*E. fetida*	+*E. fetida*	−*E. fetida*	+*E. fetida*	−*E. fetida*	+*E. fetida*	E		PS		E × PS	
									F	P	F	P	F	P
TR	22.71 ± 1.11	27.86 ± 0.51	25.57 ± 1.09	28.00 ± 0.90	17.86 ± 0.70	21.43 ± 0.72	19.71 ± 0.92	24.00 ± 0.69	40.97	0.000	28.93	0.000	0.91	0.444
TN	622.00 ± 42.87	454.29 ± 26.78	961.29 ± 70.00	523.14 ± 26.51	566.43 ± 24.29	359.29 ± 24.17	925.71 ± 73.05	441.29 ± 34.30	130.03	0.000	18.24	0.000	3.97	0.013
Ba	305.62 ± 25.12	233.92 ± 20.56	565.58 ± 42.81	158.74 ± 12.33	186.07 ± 12.09	121.93 ± 9.33	468.73 ± 40.32	208.83 ± 25.56	121.42	0.000	27.35	0.000	12.31	0.000
Fu	48.52 ± 4.75	35.71 ± 5.40	112.67 ± 11.10	127.9 ± 16.04	108.6 ± 19.33	65.17 ± 9.35	158.08 ± 18.81	48.39 ± 4.77	14.20	0.000	17.88	0.000	9.07	0.000
Pp	220.63 ± 22.24	144.3 ± 20.85	202.96 ± 39.01	159.22 ± 15.43	171.69 ± 19.57	135.04 ± 17.28	258.2 ± 26.47	160.77 ± 11.35	15.38	0.000	2.00	0.126	0.77	0.516
Om	47.23 ± 5.30	40.36 ± 3.86	80.08 ± 14.78	77.28 ± 10.38	100.07 ± 11.74	37.15 ± 4.06	40.77 ± 6.19	23.32 ± 2.65	15.88	0.000	15.79	0.000	4.89	0.005

Data are means ± SE, n = 7. SN, soil nematode; TR, total genus richness; TN, total soil nematode; Ba, bacterivores; Fu, fungivores; Pp, plant parasite; Om, omnivores-predators; E, earthworms; PS, plant species; E × PS, earthworms × plant species interaction.

**Figure 2:**
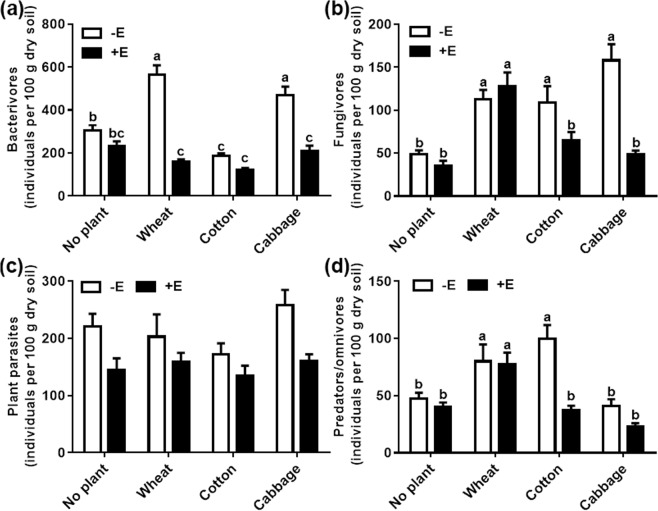
Abundances of four nematode trophic groups (**a–d**) (individuals per 100 g dry soil) under no plant and three plant species with or without added earthworms. All valued are means + SE, n = 7. Differences letters indicate significant differences between treatments (Tukey HSD test).

Earthworm addition reduced the abundance of bacterivores in wheat and cabbage (Fig. 2a, Table 3). Furthermore, significant decrease in abundance of fungivores was also observed in cotton (39.99%) and cabbage (69.39%) (Fig. 2b and Table 3) when compared with no addition of earthworm. However, there was no significant difference with plant parasites among no plant and three different plant species (Table 3, Fig. 2c). Finally, we found the abundance of omnivores-predators was remarkably decreased by earthworm addition in cotton (Table 3, Fig. 2d).

Compared with the no plant without added earthworm treatments, wheat and cabbage had the higher abundance of total nematode (Fig. 1a), bacterivores (Fig. 2a) and fungivores (Fig. 2b) and cotton had the higher abundance of fungivores (Fig. 2b) and omnivore-predators (Fig. 2d). However, the abundance of the total nematodes, bacterivores, fungivores and omnivore-predators had no significant difference compared with in the no plant with added earthworm treatments under the three plant species treatments except for in the wheat treatments.

The maximum and minimum relative bacterivore abundances were found in the wheat without and with added earthworm treatments, respectively. Earthworms decreased the relative abundance of fungivores except for in wheat but increased the relative abundance of plant parasites in wheat, cotton and cabbage. Except for in cotton, the relative abundance of omnivore-predator was enhanced by earthworm activity (Table 4).

**Table 4:** Mean values of nematode trophic groups in no plant and three different agricultural plant species (PS) and earthworm treatment (E) and the results of two-way ANOVA on the E and PS on the proportion of bacterivores (Ba%), Fungivores (Fu%), Plant Parasite (Pp%), and Omnivores-predators (Om%).

Trophic group		Ba%	Fu%	Pp%	Om%
Treatment					
No plant	−*E. fetida*	51.43 ± 3.29	8.14 ± 1.49	35.29 ± 1.81	7.71 ± 0.92
	+*E. fetida*	49.00 ± 1.88	8.00 ± 0.87	31.43 ± 3.41	9.00 ± 0.90
Wheat	−*E. fetida*	59.14 ± 2.79	11.71 ± 0.75	20.57 ± 2.67	8.57 ± 1.62
	+*E. fetida*	30.57 ± 2.20	24.14 ± 1.99	30.58 ± 3.15	14.72 ± 1.78
Cotton	−*E. fetida*	33.29 ± 2.89	18.86 ± 2.89	30.43 ± 3.22	17.43 ± 1.53
	+*E. fetida*	34.00 ± 1.85	18.00 ± 1.79	37.14 ± 3.23	10.86 ± 1.56
Cabbage	−*E. fetida*	50.71 ± 2.63	17.11 ± 1.81	27.82 ± 1.74	4.36 ± 0.56
	+*E. fetida*	46.57 ± 3.39	10.38 ± 0.72	37.14 ± 2.95	5.29 ± 0.52
*E. fetida* (E)		*	ns	*	ns
Plant species (PS)		*	*	ns	*
(E × PS)		*	*	ns	*

*P < 0.01 indicate significant differences; ns, not significant. Values are means ± SE, n = 7.

### Nematode community diversity and ecological indices

Two-way ANOVA showed that earthworm addition and plant species significantly affected the nematode community indices *H*′, SR, ΣMI and NCR (P < 0.01) but not *J*′. The values of λ were significantly influenced by earthworm activity (P < 0.01). The interactive effects earthworms and plant species was identified for the values of *H*′, *J*′, λ, ΣMI, and NCR (Table 5).

**Table 5:** Changes in the values of nematode ecological indices under no plant and three plant species with or without added earthworms and effect of earthworms (E), plant species (PS) and earthworms × plant species interaction (E × PS) on soil nematode ecological indices. *H*′, Shannon-Wiener Index; SR, Species Richness; *J*′, Evenness Index; λ, Simpson’s Index for Dominance; NCR, Nematode Channel Ratio; ΣMI, The Modified Maturity Index.

Indices	No plant		Wheat		Cotton		Cabbage		Results of two-way ANOVA					
	−*E. fetida*	+*E. fetida*	−*E. fetida*	+*E. fetida*	−*E. fetida*	+*E. fetida*	−*E. fetida*	+*E. fetida*	E		PS		E × PS	
									F	P	F	P	F	P
*H*′	2.68 ± 0.04^b^	3.03 ± 0.05^a^	2.88 ± 0.06^a^	2.95 ± 0.03^a^	2.59 ± 0.05^b^	2.74 ± 0.04^b^	2.66 ± 0.05^b^	2.81 ± 0.04^b^	33.55	0.000	13.37	0.000	4.36	0.009
SR	3.38 ± 0.15^b^	4.44 ± 0.09^a^	3.59 ± 0.18^b^	4.32 ± 0.15^a^	2.67 ± 0.12^c^	3.48 ± 0.14^b^	2.75 ± 0.14^c^	3.79 ± 0.12^ab^	83.01	0.000	19.92	0.000	0.60	0.620
*J*′	0.86 ± 0.00^b^	0.91 ± 0.01^a^	0.89 ± 0.00^ab^	0.89 ± 0.00^ab^	0.90 ± 0.01^ab^	0.89 ± 0.01^ab^	0.89 ± 0.01^ab^	0.88 ± 0.01^ab^	2.25	0.140	0.60	0.616	4.75	0.006
λ	0.10 ± 0.01^a^	0.07 ± 0.00^b^	0.08 ± 0.01^b^	0.07 ± 0.00^b^	0.09 ± 0.00^ab^	0.07 ± 0.00^b^	0.09 ± 0.00^ab^	0.08 ± 0.00^ab^	26.28	0.000	1.83	0.155	4.16	0.011
∑MI	2.43 ± 0.03^ab^	2.42 ± 0.04^ab^	2.25 ± 0.04^b^	2.55 ± 0.04^a^	2.50 ± 0.07^a^	2.52 ± 0.04^a^	2.17 ± 0.04^b^	2.27 ± 0.04^b^	11.49	0.001	17.55	0.000	5.68	0.002
NCR	0.85 ± 0.02^a^	0.86 ± 0.02^a^	0.83 ± 0.01^a^	0.56 ± 0.03^c^	0.64 ± 0.04^b^	0.66 ± 0.02^b^	0.75 ± 0.03^ab^	0.80 ± 0.02^a^	7.31	0.010	26.93	0.000	17.75	0.000

Data are means ± SE, n = 7. P > 0.05, not significant; P < 0.01, difference significant; P < 0.05, different significant. Means not sharing the same superscript letter were statistically different at P-value of 0.05 (Tukey HSD test).

### Relationships among nematode abundances, plant species and soil environmental parameters

**Figure 3:**
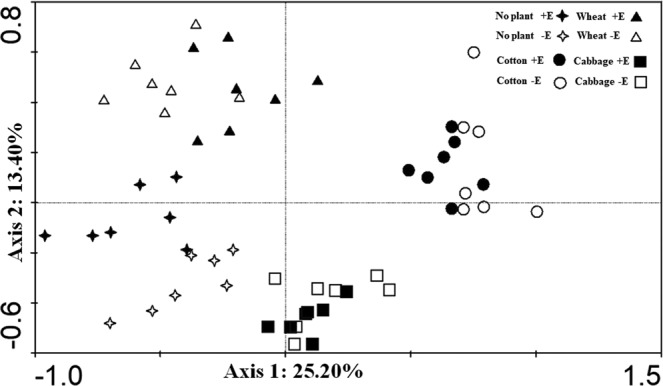
Principle component analysis (PCA) of soil nematode communities under no plant and three plant species with or without added earthworms. −E, without earthworms; +E, with earthworms.

PCA (Fig. 3) showed that the nematode community compositions in no plant, wheat, cotton and cabbage were distinguished by the fist canonical axis, which explained 25.20% of the total variation. The distances between samples under the 3 plant species treatments were longer than that between with and without added earthworm treatments, which indicated plant species had more effects on soil nematode community composition than earthworm addition. The second axis explained 13.40% of the total variation.

**Figure 4:**
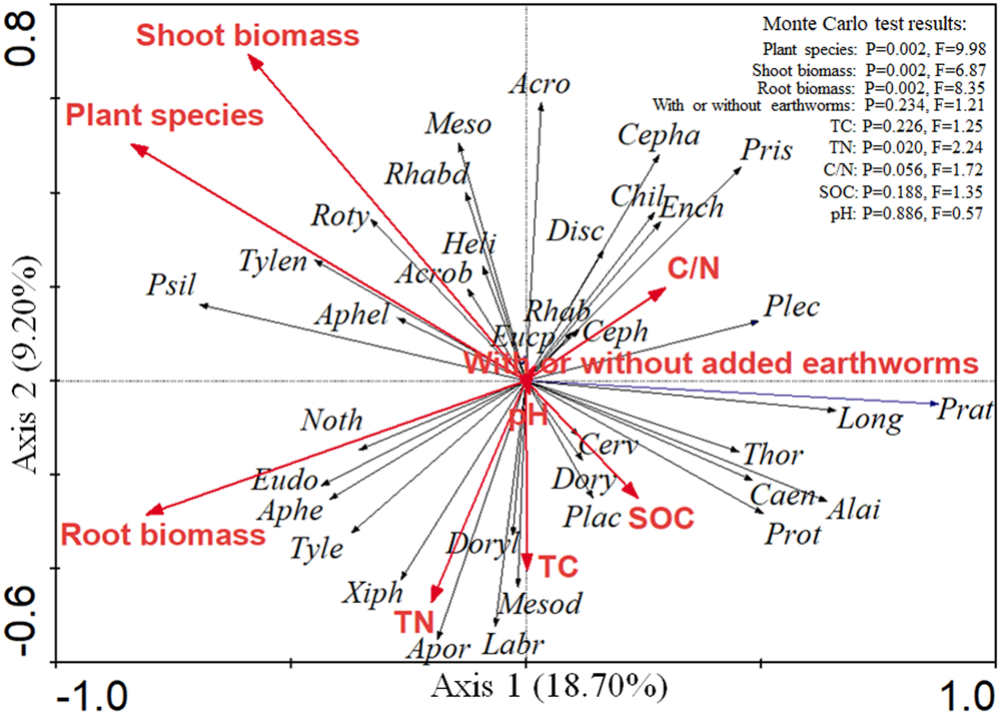
Redundancy analysis (RDA) diagram of soil nematode genera, soil properties, and treatment variables (plant species, with or without added earthworms, shoot biomass, root biomass). *Ceph*, *Cephalobus*; *Eucp*, *Eucephalobus*; *Acro*, *Acrobeloides*; *Chil, Chiloplacus; Cerv, Cervidellus; Acrob*, *Acrobeles*; *Rhab*, *Rhabditis*; *Prot, Protorhabditis*; *Alai*, *Alaimus*; *Plac*, *Placodira*; *Caen*, *Caenorhabditis*; *Plec*, *Plectus; Pris*, *Prismatolaimus*; *Meso*, *Mesorhabditis*; *Rhabd, Rhabditophanes*; *Aphe*, *Aphelenchoides*; *Tyle*, *Tylencholaimus*; *Aphel*, *Aphelenchus*; *Cepha*, *Cephalenchus*; *Tylen*, *Tylenchus*; *Heli, Helicotylenchus*; *Roty*, *Rotylenchus*; *Psil*, *Psilenchus*; *Noth*, *Nothotylenchus*; *Xiph*, *Xiphinema*; *Prat*, *pratylenchus*; *Eudo*, *Eudorylaimus*; *Thor*, *Thorneella*; *Disc, Discolaimus*; *Dory*, *Doryllium*; *Mesod*, *Mesodorylaimus*; *Apor*, *Aporcelaimus*; *Ench*, *Enchodelusthorne*; *Doryl*, *Dorylaimus*; *Labr*, *Labronema*. TC: total carbon; TN: total nitrogen; C/N, total carbon/total nitrogen; SOC: soil organic carbon.

Based on the different community composition of soil nematodes under no plant and 3 different plant species, RDA (Fig. 4) was performed to analyze the relationship between nematodes and environmental properties. Results showed that eigenvalues were 0.187 (F = 3.069, P = 0.002) and 0.092 for axis 1 and axis 2, respectively, and the first two axes explained 27.90% of species-environment variation. The interpretation amount of plant species, root biomass, shoot biomass and earthworm addition accounted for 41.60%, 21.33%, 12.80% and 6.13% of the explanation variations of all environmental factors in soil nematode compositions, respectively. The soil TN explained 4.80% of the variation in soil nematode community (Fig. 4).

### Soil physical and chemical properties

Two-way ANOVA showed that the presence of earthworms increased the soil TC, and SOC values. Significant differences in the values of TC, TN, and C/N were observed among the different plant species (Table 6). No significant interaction effect on soil properties was found between earthworm addition and plant species.

**Table 6:** Changes in the values of soil physicochemical properties under no plant and three plant species with or without added earthworms and effect of earthworms (E), plant species (PS) and earthworms × plant species interaction (E × PS) on index of soil physicochemical properties. TC (g/kg), Total Carbon; TN (g/kg), Total Nitrogen; SOC (g/kg), Soil Organic Carbon; C/N, The Ratio of Total Carbon/Total Nitrogen.

Indices	No plant		Wheat		Cotton		Cabbage		Results of two-way ANOVA					
	−*E. fetida*	+*E. fetida*	+*E. fetida*	−*E. fetida*	−*E. fetida*	+*E. fetida*	−*E. fetida*	+*E. fetida*	E		PS		E × PS	
									F	P	F	P	F	P
TC	1.55 ± 0.03^ab^	1.57 ± 0.03^ab^	1.51 ± 0.01^ab^	1.57 ± 0.03^ab^	1.53 ± 0.01^ab^	1.61 ± 0.02^a^	1.48 ± 0.02^b^	1.50 ± 0.01^b^	7.22	0.010	4.70	0.006	0.86	0.467
TN	0.07 ± 0.00^b^	0.08 ± 0.00^ab^	0.08 ± 0.00^ab^	0.08 ± 0.00^ab^	0.08 ± 0.00^ab^	0.08 ± 0.00^a^	0.07 ± 0.00^b^	0.07 ± 0.00^b^	2.67	0.109	5.29	0.003	1.22	0.313
SOC	0.49 ± 0.02	0.51 ± 0.02	0.47 ± 0.02	0.48 ± 0.02	0.44 ± 0.01	0.52 ± 0.01	0.43 ± 0.02	0.46 ± 0.04	4.15	0.047	1.90	0.142	0.72	0.546
C/N	21.36 ± 0.65	20.96 ± 1.03	18.95 ± 0.53	19.70 ± 0.54	19.89 ± 0.37	19.11 ± 0.35	20.02 ± 0.42	20.27 ± 0.47	0.01	0.910	4.08	0.012	0.67	0.573
pH	8.42 ± 0.05	8.49 ± 0.04	8.42 ± 0.06	8.46 ± 0.06	8.41 ± 0.04	8.47 ± 0.06	8.40 ± 0.04	8.45 ± 0.05	1.79	0.187	0.13	0.944	0.02	0.995

Data are means ± SE, n = 7. P > 0.05, not significant; P < 0.01, difference significant; P < 0.05, different significant. Means not sharing the same superscript letter were statistically different at P-value of 0.05 (Tukey HSD test).

## Discussion

### Influence of earthworm presence

This study demonstrated epigeic earthworm addition suppressed the soil nematode abundance, which is consistent with previous reports^9,10,14,17,42^ and demonstrated partly our first hypothesis “earthworm presence may decrease nematode abundance under planting crops”. Direct grazing by earthworms causes a decline in nematode numbers, as indicated by the presence of living and dead nematodes^43^ and nematode cuticles^44^ in the digestive systems of earthworms. The second reason may be that the increasing content of soil total carbon and organic carbon by earthworm addition had the negative effects on the abundance of soil nematode. Another reason was that earthworms and nematodes competed for food resources, which resulted in the decreasing nematodes. Except for in wheat, the total genera richness was increased by earthworm activity, indicating a shift of soil food web to a relatively complex community.

Earthworm addition suppressed the bacterivore abundance in wheat and cabbage but not in cotton, which may be related to the important role of plant species in the bacterial composition of the rhizosphere^45^. As soil passes through the earthworm gut, the suppressing effects have been shown to be selective towards soil bacteria^46,47^; In fact, decreases in the bacterial biomass have been found in three types of animal manure after transit through the earthworm gut^48^. We found that the fungivore abundance was lower in cotton and cabbage in the presence compared with the absence earthworms. In this study, the mean abundances of two fungivores genera (*Aphelenchoides* and *Aphelenchus*) were decreased in cotton and cabbage by earthworm activity. However, this change was not observed in the fungivore abundances in wheat (Fig. 2b), which may be due to a high number of fungal pathogens found in continuous cropping wheat^49^. Microorganisms, especially fungi, might be the main constituents of the epigeic earthworm diet^50^. A decrease in the abundance of fungivores in cotton and cabbage was attributed to the transit of soil through the earthworm gut. We speculated that the increase of the fungi number in the wheat treatment counteracted the quantity of earthworms ingesting the fungi, which led to the lack of change in the abundance of fungivores. However, the results of this study were not consistent with reports that showed an increasing abundance of fungal populations in the presence of earthworms^12,15^. The abundance of plant parasites was not affected by earthworm addition which was consistent with the study^51^. The omnivore-predator abundance in cotton with earthworms was lower than that without earthworms. Specifically, the mean abundances of *Dorylaimus*, *Eudorylaimus* and *Labronema* were depressed in cotton (from 17.43, 30.57 and 20.23 individuals/100 g dry soil to 8.00, 3.43 and 8.33 individuals/100 g dry soil in the absence and presence of earthworms, respectively), which suggested that these genera were possibly directly ingested by earthworms.

Except for cotton, the relative abundance of bacterial feeders decreased in all treatments when the earthworms were present. Such a pattern could be ascribed to the decreasing percent of the two dominant genera *Cephalobus* and *Rhabditis* in no plant and wheat; and the decreasing percent of the genera *Acrobeles* and *Rhabditis* in cabbage. The lower relative abundance of plant parasites in the presence of earthworms was ascribed to the decreasing relative abundance of the genera *Cephalenchus* and *Pratylenchus* in no plant; the increasing relative abundance the genera *Cephalenchus*, *Helicotylenchus* and *Nothotylenchus* in the presence of earthworms resulted in the higher relative of abundance of plant parasites under the three different plant species. In addition, earthworms increased the relative abundance of the genera *Tylenchus* and *Pratylenchus* in wheat, and enhanced the relative abundance of the genera *Cephalenchus* and *Xiphinema* in cotton, and increased the relative abundance of the genera *Tylenchus* and *Rotylenchus* in cabbage.

The higher *H*′ and SR in the presence of earthworms indicated a high number of soil nematode genera and a more stable soil nematode community structure, respectively. The lower λ values also showed the diversity of soil nematode community structure. The increasing values of ΣMI suggested that the nematode community structure was better and the soil food web complexity was increased under the earthworm treatment. The increase in the NCR value under earthworm addition suggested that earthworms increased the contribution of the bacterial decomposition channels in the soil food web (Table 5). However, the lower NCR value in wheat was ascribed to the higher fungivore abundance in the presence of earthworms (mean 127.89 individuals/100 g dry soil) when compared to that without earthworms (112.67 individuals/100 g dry soil).

### Effects of different agricultural plant species

In the absence of earthworms, the bacterivore abundance showed varying responses to the three different plant species compared with no plant, which may related to different plant species supporting specific bacterial communities due to variations in the spectra of root exudates^52–55^. The fungivorous abundance appeared to be influenced by different plant species. In the soil, fungal-feeding nematodes can feed on saprophytic, pathogenic and mycorrhizal fungi. Unfortunately, we do not have any data on the number of mycorrhizal hyphae. Differences in the omnivore-predators abundance may be explained by differences in food sources. The different *H*′, SR, λ, ∑MI and NCR values were ascribed to the different soil nematode community compositions (Fig. 3) under different agricultural plant species. Reasons may be related to the effects of different root exudates from plant species on the soil nematode population. Furthermore, RDA revealed plant species could explain the higher percent of total variance of soil nematode community than earthworm addition which indicated the plant species effects on the soil nematode community composition were more significant than earthworm presence and answered our second hypothesis.

### Interactive effects of plant species and earthworms on soil nematodes

Compared with the no plant with added earthworm treatments, the abundance of total nematode, bacterivores, fungivores and omnivore-predators under 3 agricultural plant species were weakened (Figs 1 and 2); whereas the abundance of fungivores, and omnivore-predators in wheat was not decreased and verified partly our first hypothesis “such effect may be enhanced in a system where earthworms stimulated plant growth”. These indicated in the wheat system treatments the mechanism of earthworm effects on the fungivores and omnivore-predators was not different from that in the cotton and cabbage treatments. The co-presence of both earthworms and plant species in soils could significantly affect the abundance of total soil nematode, bacterivores, fungivores and omnivore-predators. This is consistent with our third hypothesis “the interaction effects between earthworms and plant species would affect the soil nematode abundance”. We speculated that the system where earthworms stimulated plant growth may enhance the interaction effects. Earthworms significantly increased the shoot biomasses of wheat and cotton, the biomass of cabbage and the root biomass of wheat and cotton (Table 1). The enhancing plant biomasses in the presence of earthworms might have the effects on the abundance of total nematodes, bacterivores, and fungivores resulting in the decreasing abundance of soil nematode. In addition, significant different in the diversity and ecological indices *H*′, *J*′, λ, ∑MI, and NCR under the earthworm addition and different plant species treatments which was consistent with our third hypothesis were likely to related to the enhancing effects of earthworms in the presence of plants. Obviously, more studies are required to determine the complex interplay between earthworms and plants. The results obtained in the present study proposed the necessity that we should broaden the ecological context of soil biota by considering the interactive effects of plants and earthworms on the soil nematode community.

## Conclusions

In conclusion, earthworm presence weakened the crop species effects on soil nematode abundance in the wheat, cotton and cabbage treatments. Plant species were crucial for the distribution of soil nematode communities. The interaction effects of earthworms and plant species changed the trophic structure of the soil nematode community, and earthworms mostly decreased the soil nematode abundance but significantly increased the values of the soil nematode community structure indices. The effects of plant species on the soil nematode community composition were more significant than the effects of earthworm addition.

Overall, the present study provides insights into the interaction effects of earthworms and plants on soil nematode communities. Future studies should determine whether the effects of earthworms on soil nematode community structure are modified through plants. Ultimately, this knowledge will help us to better understand the interplay among plants, earthworms, soil microorganisms, and soil nematodes.
